# Novel silver metformin nano-structure to impede virulence of *Staphylococcus aureus*

**DOI:** 10.1186/s13568-022-01426-6

**Published:** 2022-06-30

**Authors:** Hisham A. Abbas, Ghada H. Shaker, Farag M. Mosallam, Salwa E. Gomaa

**Affiliations:** 1 Department of Microbiology and Immunology, Faculty of Pharmacy, Zagazig University,, Zagazig, Egypt; 2 Drug Microbiology Lab., Drug Radiation Research Department, Biotechnology Division, National Center for Radiation Research and Technology (NCRRT), Egyptian Atomic Energy Authority, Cairo, Egypt

**Keywords:** Multidrug resistant *S. aureus*, Silver metformin nanostructure, Virulence, Quorum sensing inhibition

## Abstract

*Staphylococcus aureus* is a prevalent etiological agent of health care associated and community acquired infections. Antibiotic abuse resulted in developing multidrug resistance in *S. aureus* that complicates treatment of infections. Targeting bacterial virulence using FDA approved medication offers an alternative to the antibiotics with no stress on bacterial viability. Using nanomaterials as anti-virulence agent against *S. aureus* virulence factors is a valuable approach. This study aims to investigate the impact of metformin (MET), metformin nano (MET-Nano), silver metformin nano structure (Ag-MET-Ns) and silver nanoparticles (AgNPs) on *S. aureus* virulence and pathogenicity. The in vitro results showed a higher inhibitory activity against *S. aureus* virulence factors with both MET-Nano and Ag-MET-Ns treatment. However, genotypically, it was found that except for *agrA* and *icaR* genes that are upregulated, the tested agents significantly downregulated the expression of *crtM*, *sigB*, *sarA* and *fnbA* genes, with Ag-MET-Ns being the most efficient one. MET-Nano exhibited the highest protection against *S. aureus* infection in mice. These data indicate the promising anti-virulence activity of nanoformulations especially Ag-MET-Ns against multidrug resistant *S. aureus* by inhibiting quorum sensing signaling system.

## Key points

- A new formation of silver metformin nanostructure.
- The in vitro inhibition of *S. aureus* virulence factors.
- Nano structure form improves the activity of anti-virulence agents.

## Introduction

*S. aureus* is a common causative agent of healthcare associated infections as well as community acquired infections, including soft-tissue, cutaneous infections and severe systemic infections (Abbas et al. 2017, 2019; de Lencastre et al. 2007). *S. aureus* has an arsenal of virulence factors that helps the microorganism evade the immune system and cause disease in host. This includes adhesins (protein A and fibronectin binding protein), secreted enzymes (protease, lipase), secreted toxins (Panton Valentine leukocidin and hemolysins), biofilm formation and staphyloxanthin production (Gordon et al. 2013; Gould et al. 2012; Pereira et al. 2009). Improper antibiotic dispensing policy enabled the spread of antibiotic resistant infections (Boucher et al. 2009; Nitsch-Osuch et al. 2015). *S. aureus* is one of the superbugs that show high rates of multidrug resistance (de Lencastre et al. 2007). Development of new antibiotics takes a long time and demands excellent economic efficiency. In addition, fast resistance progress shortens their lifespan (Boucher et al. 2013; Fernandes and Martens 2017).

Instead of targeting cellular growth of bacteria by antibiotics, using anti-virulence drugs can eradicate pathogens without exerting cidal effect on them (Finlay and Falkow 1997; Rasko and Sperandio 2010). The use of FDA approved drugs is beneficial in terms of available safety and pharmacokinetic characteristics data, short time and cost needed to produce novel drugs (Miro-Canturri et al. 2019; Mullard 2012). There are several anti-virulence therapy approaches available, with quorum sensing being one of the most commonly investigated (LaSarre and Federle 2013).

Quorum sensing (QS) is the fundamental regulator of bacterial virulence. Several regulatory loci regulate the development of *S. aureus* virulence factors. They include sigma factor *σ*^*B*^ (encoded by *SigB* gene), staphylococcal accessory regulator (*sarA*) and accessory gene regulator (*agr*). QS in *S. aureus* is regulated by *agr* operon. It is responsible for upregulation of the genes encoding superantigens, cytotoxins, and secreted enzymes. *SigB* gene is involved in induction of many stress genes and partially in expression of *SarA* and production of staphyloxanthin (Bien et al. 2011; Burnside et al. 2010).

Dehydrosqualene synthase (CrtM) enzyme, encoded by *crtM* gene, catalyzes the first step in staphyloxanthin biosynthesis (Song et al. 2009). The fibronectin-binding proteins encoded by *fnbAB* genes function as adhesins and invasins to regulate attachment and internalization and facilitate biofilm assembly (Houston et al. 2011; O'Neill et al. 2008). In addition, it was reported that the major component that affects the biofilm maturation in *S. aureus* is the polysaccharide intercellular adhesin (PIA). The synthesis of PIA requires four gene products. These products are encoded in the *icaADBC* operon that is negatively regulated by *icaR* gene (Lei et al. 2011).

One of the therapeutic trends required to counteract multidrug resistant (MDR) infections caused by bacteria is the use of nanoparticles that have distinct physical and chemical characteristics in comparison with their bulk matter (Wang et al. 2017). Nanomeric materials provide advantages such as higher contact between bacteria and compounds, higher cell permeability and improved absorption and bioavailability (Jamil and Imran 2018; Zaidi et al. 2017). In order to synthesize nanomaterials, physical, chemical, and biological methods are used (Kaur 2018). However, the radiation method, as compared to traditional methods is simpler and can provide products in a totally reduced, high purity and ultra-stable condition. In addition, no excessive oxidation products are produced and therefore excess reducing agents are not required (Remita et al. 1996).

Metformin is a common biguanide hypoglycemic medication. It was reported that metformin possesses antibacterial and anti-virulence activities against *P. aeruginosa* (Hegazy et al. 2020; Nasrin 2014). Silver is an old remedy with antibacterial broad spectrum and low toxicity (Chen and Schluesener 2008). Moreover, silver nanoparticles (AgNPs) have a wide variety of antibacterial, antifungal and antiviral activities in addition to anti-virulence activities (Li et al. 2020; Loo et al. 2016; Murphy et al. 2015; Qais et al. 2021).

This research study was performed aiming to assess metformin’s anti-quorum sensing activity and investigate if its nanoform could be more efficient than the bulk. Additionally, examination of the potential for reducing virulence factors and pathogenicity of *S. aureus* using a combination of metformin and AgNPs was performed.

## Materials and methods

### Media and chemicals

Mannitol salt agar (MSA), tryptone soya agar (TSA), tryptone soya broth (TSB), Mueller Hinton agar (MHA) and Mueller Hinton broth (MHB) were the products of Oxoid (St. Louis, USA). Other chemicals were of pharmaceutical grade. Metformin (MET) and silver were purchased commercially from Sigma Chemical Company, St. Louis, Mo, USA.

### Bacterial isolates

In this study, *S. aureus* ATCC 6538, in addition to six clinical MDR isolates of *S. aureus* (SA2, SA8, SA9, SA12, SA14 and SA17) were used. The standard strain was kindly gifted by the stock culture collection of Microbiology and Immunology Department, Faculty of pharmacy, Al Mansoura University, while clinical isolates were refreshed from the stock culture collection of Microbiology and Immunology Department, Faculty of pharmacy, Zagazig University. These six isolates were selected based on higher production of virulence factors among 20 clinical isolates screened. They were isolated from patients suffering from burn and wound infections in addition to blood infections and pneumonia; the sources are blood (SA2), burn swab (SA8 and SA9), surgical wound swab (SA12 and SA14) and endotracheal aspirate (SA17).

To maintain the isolates, cultures were maintained in MHB with 10–15% glycerol added after growth. The cultures were kept at −80 °C.

### Nanostructure preparation and validation

The formulations of metformin nano (MET-Nano), silver metformin nanostructure (Ag-MET-Ns) and silver nanoparticles (AgNPs) were prepared as follows:

Silver nanoparticles were synthesized by mixing of silver nitrate (1 mM) solution with PVP (50 mg/mL) 1:1 v/v and maintained under stirring until a homogeneous solution was obtained followed by irradiation at 40 kGy gamma ray. Metformin nano was prepared by addition of tween 80 (10%) and isopropyl alcohol (0.02%) dropwise into aqueous metformin (100 mg/mL) under continuous stirring using homogenizer at 10000 rpm for 30 min. The solution was then sonicated using ultrasonic sonicator for 1 h. Silver metformin nanostructure was prepared by dropwise addition of silver nanoparticles colloid (1 mM, prepared previously) into a mixture of aqueous metformin (100 mg/mL), tween 80 (10%) and isopropyl alcohol (0.02%) under continuous stirring using homogenizer at 10,000 rpm for 30 min. The solution was then sonicated using ultrasonic sonicator for 1 h.

For validation of nanoformulation, the size and stability of the provided Ag-MET-Ns were characterized by using size distribution and zeta potential measurements. The Fourier transforms infrared spectroscopy (FT-IR) was used to estimate the Ag-MET-Ns function moiety (El-Batal et al. 2020). The particle sizes of Ag-MET-Ns were carried out using TEM (JEOL electron microscope JEM-100 CX) operative at 80 kV accelerating energy.

### Determination of minimum inhibitory concentrations (MICs) of the tested agents

The minimum inhibitory concentrations (MICs) of MET, MET-Nano (stock solution of 100 mg/mL, each), Ag-MET-Ns (100 mg/mL-50 ppm) and AgNPs (50 ppm) against the tested isolates were assessed using the broth micro-dilution method using 96-well microtiter plate according to the Clinical and Laboratory Standards Institute (CLSI) guidelines (Patel et al. 2015). Briefly, Bacterial isolates were grown overnight in MHA media, diluted to reach a turbidity equal to 0.5 McFarland Standard (1.5 × 10^8^ CFU/mL) with sterile saline and then were 1:100 diluted to reach 10^6^ CFU/mL with MHB. Aliquots of 50 µL of serially diluted tested agents were added to the wells of microtiter plate. The bacterial suspension was delivered in aliquots of 50 µL to the wells of microtiter plate. Following overnight incubation at 37 °C, the lowest concentrations of the tested agents that inhibit the bacterial visible growth were calculated and considered as the MIC values.

### Phenotypic assay of *S. aureus* virulence factors

#### Bioflm inhibition assay

The biofilm forming capacity of the tested clinical strains was quantitatively assayed according to Stepanović et al. (2007). The standard strain *S. aureus* ATCC 6538 was previously reported as strong biofilm former (Zhou et al. 2019). *S. aureus* suspensions were obtained from overnight cultures in TSB and diluted to reach turbidities that match 0.5 McFarland standard. The prepared suspensions were 1:100 diluted in fresh TSB supplemented with 1% glucose. Aliquots of 200 μL/well of diluted suspensions were added the wells of microtiter plates and incubated for 48 h at 37 °C. Negative-control wells containing 200 µL/well of fresh TSB supplemented with 1% glucose alone were included in each plate. Following incubation, the contents of wells were decanted and the plates were washed three times with water to eliminate planktonic cells before being air dried.

Aliquots of 150 μL/well of 99% methanol were added to fx the biofilms and left for 20 min. After that, aliquots of 150 μL/well of crystal violet (1%) were added for 15 min to stain the biofilms, washed three times with water and dried. The bound dye was solubilized with 150 µL/well of 33% glacial acetic acid. The OD_570_ was measured using spectrofluorometer (Biotek, USA). The experiment was done in duplicate. The biofilm forming capacity was assessed according to the criteria of Stepanović et al. (2007).

The biofilm inhibitory activities of the tested agents against the tested isolates were performed in the presence of 1/10 MIC of them using the same procdure. The percentage (%) of biofilm inhibition was estimated using from the following formula;

% of biofilm inhibition = [(Control _OD 570 nm_—Treated _OD 570 nm_)/Control _OD 570 nm_] × 100.

### Staphyloxanthin inhibition assay

The inhibitory activities of the tested agents against staphyloxanthin pigment were assessed (Kossakowska-Zwierucho et al. 2016). *S. aureus* isolates were grown overnight in TSB to an optical density equal 2.0 in 5 mL volume in the presence and absence of 1/10 MIC of tested agents. After incubation, bacterial cells were harvested by centrifugation at 4000 rpm at 4 °C for 10 min, washed twice with double distilled water. Pellets were then resuspended in 1.5 mL of 99% methanol, agitated for 2 h in the dark to extract pigment. After centrifugation, the cell free supernatants was measured at optical density 450 nm using spectrofluorometer (Biotek, USA). The experiment was performed in duplicate.

### RNA extraction and relative gene expression measurement in *S. aureus* using qRT‑PCR

The standard strain *S. aureus* ATCC 6538 was selected to determine the effect of the tested agents on the expression levels of QS regulatory genes (*crtM*, *sigB*, *agrA*, *sarA*, *icaR* and *fnbA*) by qRT-PCR. Primers used in this study are listed in Table 1. The standard strain was grown in TSB in the presence and absence of sub-MICs of the tested agents and overnight incubated at 37 °C. After the incubation period, cells were harvested by centrifugation and immediately stored at −80 °C. The total RNA from *S. aureus* isolate was extracted and purified using TRIzol Reagent (15596026, Life Technologies, USA) following the manufacturer protocol. Reverse trancription using QuantiTect Reverse Transcription Kit was performed for cDNA synthesis. Afterthat, the cDNA was amplified using Thermo Scientific Maximas SYBR Green/Fluorescein qPCR Master Mix. The average threshold cycle (CT) values were normalized to the housekeeping gene (*16 s rRNA*) in *S. aureus*. The relative gene expression of treated isolate was compared to that in the untreated ones according to the 2^−∆∆Ct^ method (Livak and Schmittgen 2001). The experiment was done in triplicate.

**Table 1:** Primers used in qRT-PCR

Gene	Primer sequence	References
*CrtM*	F/5'-CTGCTAATTCTATGATTGGTTGTGC-3' R/5'-TGGGAATATTATGCAGCTATMGCAG-3'	Antonic et al. 2013
*SigB*	F/5'-AAGTGATTCGTAAGGACGTCT-3' R/5'-TCGATAACTATAACCAAAGCC T-3'	Antonic et al. 2013; Lee et al. 2013
*AgrA*	F/5'-GGAGTGATTTCAATGGCACA-3' R/5'-ATCCATTTTACTAAGTCACCGATT-3'	Sambanthamoorthy et al. 2006
*SarA*	F/5'-TCTTGTTAATGCACAACAACGTAA-3' R/5'-TGTTTGCTTCAGTGATTCGTTT-3'	Sambanthamoorthy et al. 2006
*icaR*	F/5'-TGCTTTCAAATACCAACTTTCAAGA-3' R/5'-ACGTTCAATTATCTAATACGCCTG-3'	Bai et al. 2019
*fnbA*	F/5'-AACTGCACAACCAGCAAATG-3' R/5'-TTGAGGTTGTGTCGTTTCCTT-3'	Sambanthamoorthy et al. 2006
*16 s rRNA*	F/5'-TGTCGTGAGATGTTGGG-3' R/5'-CGATTCCAGCTTCATGT-3'	Lee et al. 2013

*F* forward, *R* reverse

### In vivo mice infection

Using mice as an infection model, the effect of the tested agents on *S. aureus* ATCC 6538 pathogenicity was investigated. All procedures were carried out in compliance with the appropriate the ethical standards for animal welfare approved by The Institutional Animal Care and Use Committee, Zagazig University (ZU-IACUC), Egypt (Approval number: ZU-IACUC/3/F/114/2020). Bacterial load in mice tissues was determined as previously described with some modifications (Deshmukh et al. 2009). Overnight cultures in TSB with and without 1/10 MIC of the tested agents were prepared. Cultures were centrifuged and cell pellets were resuspended in phosphate-buffered saline (PBS) to achieve a bacterial density of 2.5 × 10^7^ CFU/mL. Thirty-five healthy albino mice (*Mus musculus*) with equal weights (15–20 g) were used. Mice were devided into seven random groups (5 mice/group). In group 1, mice were intraperitoneally injected with 100 µL of untreated bacteria in sterile PBS. In group 2, mice were injected with 100 µL of MET-treated bacteria, group 3 was injected with 100 µL of MET-Nano-treated bacteria, while group 4 was injected with 100 µL of Ag-MET-Ns-treated bacteria and group 5 was injected with 100 µL of AgNPs-treated bacteria. Two negative control groups are also included; group 6, mice were injected with 100 µL of sterile PBS and group 7, mice were left uninoculated. At room temperature, all groups received normal feeding and aeration. Mice were euthanized after 24 h, livers and kidneys were aseptically isolated, homogenized and plated for viable count that expressed as colony forming unit per gram (CFU/g).

### Statistical analysis

The effect of the tested agents on virulence factors expression was analyzed by GraphPad Prism 8 software using One Way ANOVA followed by Dunnett’s Multiple Comparison tests at *P* < 0.05 for signifcance.

## Results

### Preparation and characterization of nanoemulsions

**Fig. 1:**
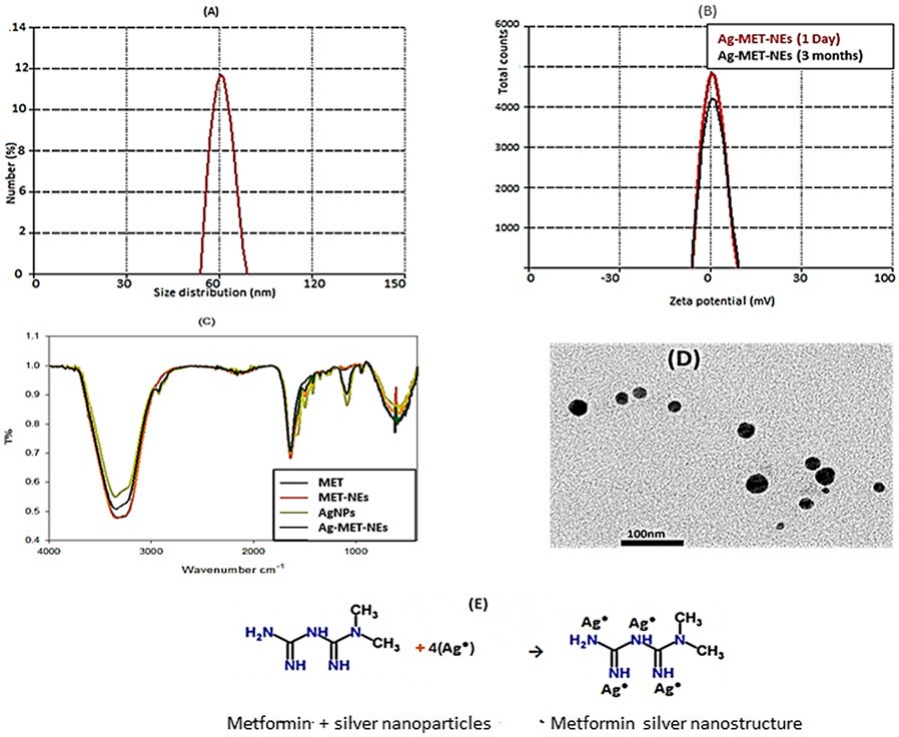
**A** Size distribution of Ag-MET-Ns at 66 nm, **B** Zeta potential of Ag-MET-Ns, **C** FT-IR of Metformin, MET-Nano, AgNPs and Ag-MET-Ns, **D** TEM image of Ag-MET-Ns and **E** image of Ag-MET-Ns stabilized form

The size distribution analysis of MET-Nano and Ag-MET-Ns was performed using DLS Zeta Sizer Technique. As shown in Fig. 1A, the size of Ag-MET-Ns, measured by DLS technique was 66 nm. Figure 1B shows that the zeta potential was at the range from −30 mV to 30 mV; an indicator of good stability of Ag-MET-Ns; no phase separation or sign of instability of samples were found. Moreover, the changes in particle size of samples initially prepared and stored samples (3 months) were not significant.

Figure 1C shows FT-IR; MET had absorption bands at 3330 cm^−1^, 1660 cm^−1^ and 620 cm^−1^. The broad peak at 3330 cm^−1^ can be specified to N–H stretching, while the band at 1660 cm^−1^ is assigned to N absorption. The band found at 617 cm^−1^ can be specified to C-H out of plane bending and C-N–C deformation. After the synthesis of Ag-MET-Ns, the decrease in the intensity of peaks may be due to physical binding of AgNPs to NH groups of MET that recommends their role in the stabilization of Ag-MET-Ns (El-Batal et al. 2018).

Figure 1D shows the TEM image of Ag-MET-Ns that confirms the spherical shape of particles with average size about 45 nm. The presence of metformin, serving as capping and stabilizing agent, (Fig. 1E) controls and prevents the aggregation and agglomeration of generated Ns.

### Minimum inhibitory concentrations (MICs) of the tested agents against *S. aureus*

The broth microdilution method was used to determine the MICs. The MICs of Metformin (MET) and Metformin nano (MET-Nano) were similar. The MIC was greatly decreased by the combination of MET and AgNPs (Ag-MET-Ns) than either MET, MET-Nano or AgNPs alone. Considering the increased sensitivity to MET, the MICs were decreased by 2–32 folds, while for MET-Nano (2–16) folds and in case of AgNPs, (4–32) folds (Table 2). The activity of the tested agents against quorum sensing and virulence factors of the tested isolates was evaluated at 1/10 MICs to avoid any effect on bacterial growth caused by these agents.

**Table 2:** MIC values of the tested agents against *S. aureus*

Tested isolates	MET (100 mg/mL)	MET-Nano (100 mg/mL)	Ag-MET-Ns (100 mg/mL–50 ppm)	AgNPs (50 ppm)
SA2	50	50	6.25	25
SA8	25	25	1.56	25
SA9	25	25	12.5	25
SA12	50	25	1.56	25
SA14	12.5	12.5	3.125	25
SA17	25	12.5	3.125	25
SA ATCC 6538	50	25	1.56	25

*MET* metformin, *MET*-*Nano* metformin nano, *Ag*-*MET*-*Ns* silver metformin nano, *AgNPs* silver nanoparticles

### Phenotypic inhibition of virulence factors of *S. aureus* by the tested agents

#### The tested agents inhibited biofilm formation

**Fig. 2:**
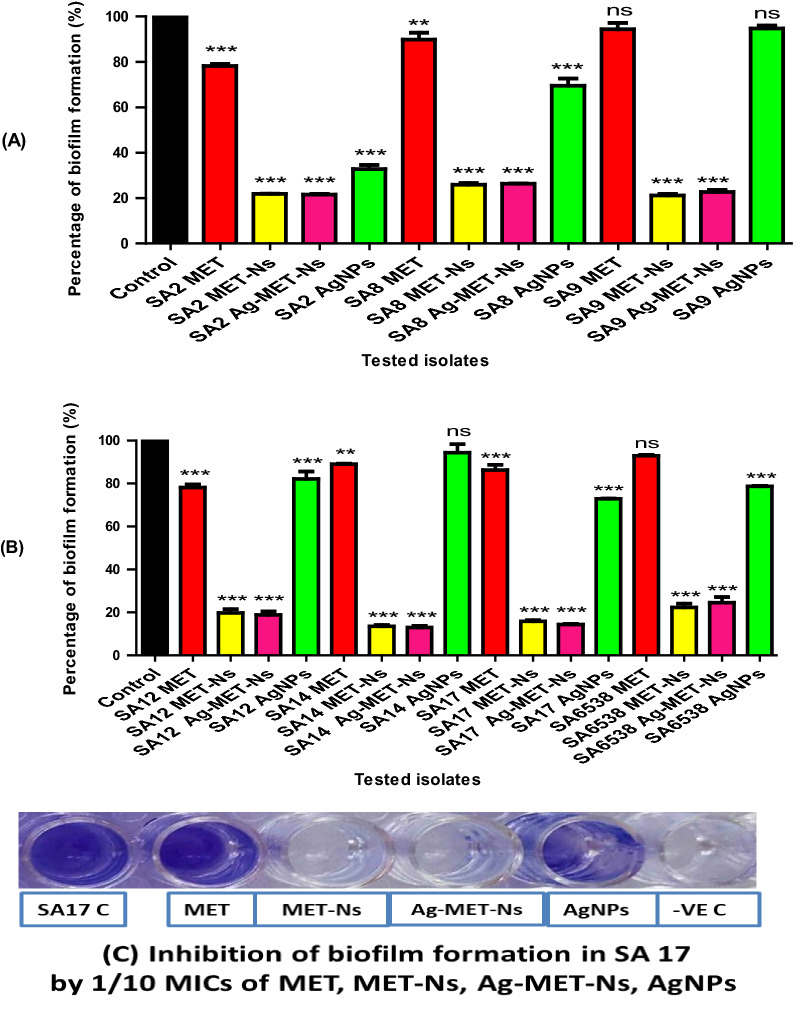
Inhibition of biofilm formation in *S. aureus* by 1/10 MICs of the tested agents. Optical density was measured at 570 nm. Significant reduction of biofilm formation was found with 1/10 MICs of the tested inhibitors in the tested bacteria compared to controls. The data shown represent the means ± standard errors. *P < 0.05, ns; non-significant

The activity of the tested agents against *S. aureus* biofilm inhibition was estimated using crystal violet assay. The tested agents significantly reduced the biofilm formation compared with controls at (*P*˂ 0.05) as shown in Fig. 2. Compared to MET that inhibited biofilm by 5.64–21.78% and AgNPs that reduced biofilm by 5.27–67.20%, MET-Nano and Ag-MET-Ns showed remarkably higher inhibitory activities (74.10–86.42% and 73.68–86.97%, respectively). No significant inhibition of biofilm formation was found with one clinical isolate (SA9) as well as standard strain when treated with MET. Also, the biofilm forming capacity was not significantly reduced in two AgNPs treated isolates (SA9 and SA14).

#### The tested agents decreased staphyloxanthin production

**Fig. 3:**
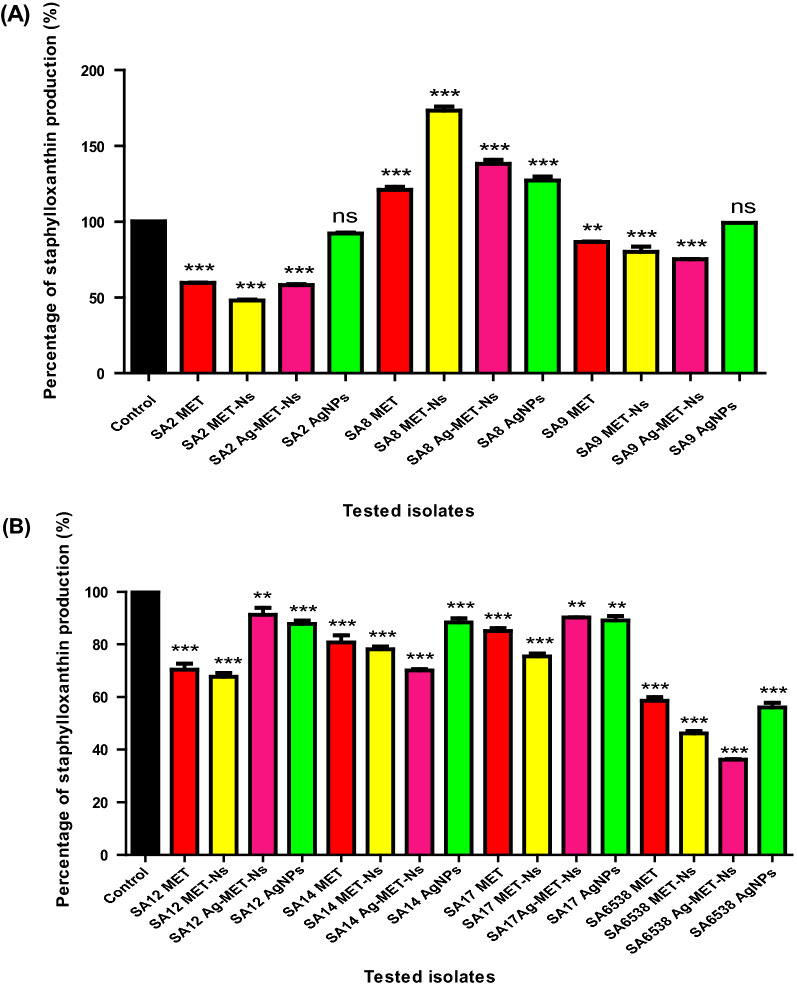
Inhibition of Staphyloxanthin pigment production by 1/10 MICs of the tested agents. The pigment was extracted with methanol from treated and untreated bacterial cells and the yellow pigment was measured at OD 450 nm. The data shown represent the means ± standard errors. *; significant *P* < 0.05, ns; non-significant

The ability of the tested agents to inhibit staphyloxanthin pigment production of *S. aureus* was estimated spectrophotometrically. The tested agents showed significant inhibition of staphyloxanthin pigment production compared to the controls at (*P* < 0.05) as shown in Fig. 3. MET-Nano and Ag-MET-Ns showed higher reduction of staphyloxanthin production (20.02–53.89% and 8.68–63.83%, respectively) than MET (13.37–41.43%) and AgNPs (0.86–43.94%). No significant inhibition of staphyloxanthin production was observed in two AgNPs treated isolates (SA2 and SA9). However, a significant increase in the production of staphyloxanthin pigment was found in one isolate (SA8) with the tested agents.

#### Estimation of the relative gene expression of QS-regulatory genes using qRT-PCR

**Fig. 4:**
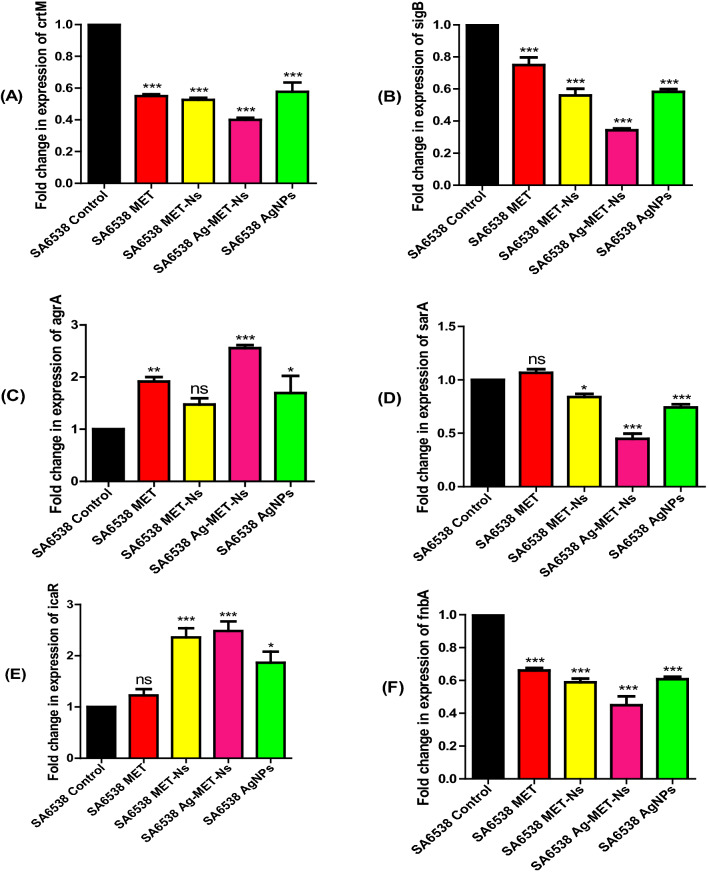
Downregulation of *S. aureus* QS genes by tested agents. **A** *crtM*, **B** *sigB*, **C** *agrA*, **D** *sarA*, **E** *icaR* and **F** *fnbA* using sub-MICs of the tested agents compared to controls. The data shown are the means ± standard errors of three biological experiments with three technical replicates each. *P < 0.05, ns; non-significant

The qRT-PCR was performed to investigate the impact of the tested agents on the expression of *S. aureus* ATCC 6538 virulence genes. The 2^−∆∆Ct^ method was used to analyze the obtained results. The tested agents significantly downregulated the expression of QS regulatory genes; *crtM*, *sigB*, *sarA* and *fnbA,* while *agrA* and *icaR* were upregulated when treated with the tested agents in comparison to the untreated controls (Fig. 4). The expression level of *crtM* was significantly reduced, the percentage reduction by MET was somehow comparable to that of MET-Nano and AgNPs (< 40%) each, whereas the highest inhibitory activity found with Ag-MET-Ns (60%). With regards to *sigB* gene expression, the lowest percentage inhibition found with MET (25%), followed by MET-Nano and AgNPs (< 40%) each, whereas Ag-MET-Ns showed the highest inhibitory activity (65.75%). In addition, Ag-MET-Ns showed the highest inhibition of *fnbA* (55.17%), while against *sarA,* the inhibitory activities of MET-Nano and AgNPs were more or less similar (16% and 25.76%, respectively), with the highest reduction observed with Ag-MET-Ns (55.15%), however, lower inhibition of 33.92%, 39.28% and 41.13% was found with MET, AgNPs and MET-Nano, respectively. On the other hand, the expression level of *agrA* was significantly increased; up to 69.4% with AgNPs, 91.7% with MET, with maximum upregulation  (155.6%) with Ag-MET-Ns, while no significant increase was observed with MET-Nano. Similarly, *icaR* expression was significantly increased in the presence of sub-MICs of the tested agents; 86.4% with AgNPs, 135.8% with MET-Nano, while Ag-MET-Ns achieved the highest percentage (148.3%). However, no significant increase was found after treatment with MET.

#### The tested agents decreased the bacterial load in liver and kidney tissues

**Fig. 5:**
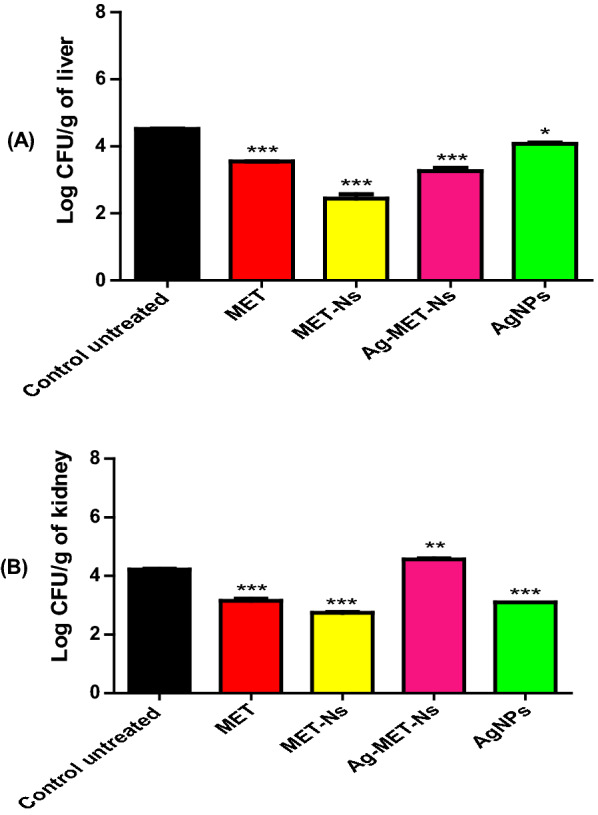
Bacterial load reduction by tested agents. *S. aureus* ATCC 6538 CFUs recovered from livers (**A**) and kidneys (**B**) of mice tissues 24 h post-infection. Bars represent mean log CFUs/g of organ tissue. The bacterial load was calculated and expressed as means ± standard errors. *P < 0.05

The bacterial load in livers and kidneys of mice treated with sub-MICs of the tested agents were significantly lower than that of the control untreated mice group (*P* < 0.05). The results were expressed as log CFU/g of organ (Fig. 5). With regards to the live bacterial counts in liver tissues, MET-Nano showed the highest protective activity with mean log reductions in viable counts decreased from 4.520 in the untreated mice group to 2.445 in MET-Nano treated mice group. However, MET and Ag-MET-Ns exhibited similar protective activities (3.545 and 3.260, respectively). The lowest protective activity was found with AgNPs (4.075). Similarly, it was found that among all tested agents, MET-Nano successfully reduced live bacterial counts in kidneys tissue. The mean log CFU reductions decreased from 4.210 in the control mice group to 2.735, 3.100 and 3.150 in MET-Nano, AgNPs and MET, respectively. A significant increase in bacterial counts was found with Ag-MET-Ns treated mice group (4.565).

## Discussion

The ability of bacteria to develop resistance to antibiotics represents a great dilemma in measures of public health authorities. This problem is heightened by the poor supply of novel antibiotics (Cegelski et al. 2008; Defoirdt 2018; Ventola 2015). Antibiotic-resistant bacteria cause severe infections as they result in high rates of infections and death; thus, new strategies to overcome this issue are required (Prestinaci et al. 2015). Drug repurposing is a substitutional way for speeding up the research and development of antimicrobials (Rangel-Vega et al. 2015; Thangamani et al. 2015).

*S. aureus* is one of *ESKAPE* pathogens that include the Gram-positive *Staphylococcus aureus* and *Enterococcus faecium* and the Gram-negative bacteria; *Klebsiella pneumoniae*, *Acinetobacter baumannii*, *Enterobacter spp*. and *Pseudomonas aeruginosa*. These bacteria are the major cause of health care associated infections over the world and exhibit potential drug resistance mechanisms (Santajit and Indrawattana 2016). Quorum-sensing regulated virulence factors are responsible for the ability of bacteria to cause infections (Grandclement et al. 2016; LaSarre and Federle 2013). Quorum sensing is therefore regarded as a valuable target for the therapeutic strategy that targets virulence.

*S. aureus* can form biofilms. These biofilms may affect human or may be formed on implants, causing persistent recalcitrant infections because of severe antibiotic resistance that may reach 1000 times higher than in planktonic cells (Mah and O'Toole 2001; Otto 2008). This may be due to impermeability of the biofilm matrix; an obstacle that could be overcome by anti-biofilm nanostructures (Ansari et al. 2014; Shah et al. 2013). In addition, *S. aureus* produces staphyloxanthin, a carotenoid pigment that reacts with the reactive oxygen species (ROS) that are liberated inside neutrophils and macrophages, and thus deactivate it, giving *S. aureus* the merit of evading innate immunity (Clauditz et al. 2006; Liu et al. 2005).

Nanoparticles are becoming increasingly popular as antimicrobial agents and are being used in a variety of applications. With increasing rates of biofilm-mediated antibiotic resistance, the antimicrobial activity of silver, especially in their nanoform has becoming a topic of research (Murphy et al. 2015). It is hypothesized that low resistance to Ag is attributed to the fact that Ag^+^ ions work on many bacterial sites at the same time. Therefore, the use of silver (Ag)-based compounds is on the rise because of silver broad spectrum of action and little likelihood of developing bacterial resistance in comparison to current antibiotic regimens (Feng et al. 2000). Moreover, many nanomaterials were found to have anti-virulence activity against *S. aureus* (Hamida et al. 2020; Salunke et al. 2014).

Metformin is a widely prescribed oral hypoglycemic over the world (Agarwal et al. 2014). In the current research study, the ability of the tested agents to target quorum sensing and to attenuate virulence was evaluated against *S. aureus* using sub-MICs (1/10) to ensure that the inhibitory effect of the tested agents is due to inhibition of bacterial virulence factors instead of affecting their growth.

In the present study, nano formulation were characterized by measurement of size distribution and zeta potential; the zeta potential value can be related to the stability of nano formulation molecules and particles that are small enough. A high zeta potential will confer stability. On the other hand, low zeta potential indicates that the attraction exceeds the repulsion resulting in breaking out of the emulsion and flocculation. In this study, the zeta potential was high. As a result, the prepared nano formulation are stable (Jadhav et al. 2015).

In the current study, it was found that MET-Nano showed similar antibacterial activity to MET against *S. aureus*. However, the combination of MET and AgNPs (Ag-MET-Ns) exhibited higher synergistic activity than that of MET-Nano or AgNPs alone (Table 2). Li et al. (2020) reported similar findings; the antibacterial activity of a novel nanoparticle known as FTP NPs that is composed from biguanide-based polymetformin (PMET), tannic acid and F-127 surfactant was similar to that of PMET alone against the test microorganisms. Furthermore, the activity of polyhexamethylene biguanide (PHMB)-functionalized silver nanoparticles conjugates against *E. coli* was investigated, and it was observed that PHMB increased AgNPs’ antimicrobial activities by almost 100 times, when compared to prior studies of AgNPs (Ashraf et al. 2012). Moreover, Yi et al*.* found that AgNPs-PHMB had a higher bactericidal effect against *S. aureus* than AgNPs and PHMB alone (Yi et al. 2019).

In the present study, it was found that MET-Nano or Ag-MET-Ns demonstrated high synergistic activities against *S. aureus* biofilm, and they were more effective than either MET or AgNPs alone. Abbas et al. (2017) reported higher reduction of PAO1 biofilm formation by metformin (67.9%). Metformin also improved the antibacterial and biofilm eradication properties of gold nanoparticles (Rasko and Sperandio 2010). Moreover, a study conducted by Li et al*.* showed that FTP NPs were more active against MRSA USA300 biofilm than PMET as shown by reduction in the bacterial cell counts by approximately 100-fold (Li et al. 2020). In addition, various investigations have shown that polyhexamethylene biguanide (PHMB) has antibacterial and biofilm inhibiting activities against a number of bacterial species (Kamaruzzaman et al. 2017; Lefebvre et al. 2018). Moreover, various research studies demonstrated contradictory findings on another biguanide compound (chlorhexidine). *S. aureus* biofilm was significantly reduced by chlorhexidine depending on concentration and the contact time in a study reported by Abdallah and Abakar (2017), however, in our study, we studied the effect of the tested agents on mature biofilm at 1/10 MIC value of each agent. In contrast to the study of Abdallah and Abakar (2017), no bactericidal activity against *S. aureus* biofilm was reported with chlorhexidine in another investigation (Vestby and Nesse 2015). Furthermore, Courrol et al. (2019) found that tryptophan silver nanoparticles (TrpAgNPs) remarkadly reduced *S. aureus* biofilm formation. In another study reported by Ali et al. (2015), significant biofilm inhibition was observed in *S. aureus* biofilms using biosynthesized AgNPs. Also, Mohanty et al. (2012) found that the treatment of *S. aureus* biofilm with AgNPs resulted in strong biofilm inhibition.

In the current study, low inhibiting activities of staphyloxanthin pigment were found with the tested agents. It was found that MET-Nano showed the highest staphyloxanthin inhibiting activity followed by Ag-MET-Ns, MET and AgNPs. Surprisingly, the tested agents increased staphyloxanthin production in one tested isolate (SA8). These results suggest further studies to investigate the mechanism of action of such formulations. The variation in the results may be attributed to the fact that staphyloxanthin can reduce the fluidity of cell membrane and stabilize its structure, modifying its chemical composition and its function (Popov Iu et al. 1976). Moreover, staphyloxanthin production is a complex process regulated by many genes such as staphyloxanthin biosynthesis operon *crtOPQMN*, *sigB* whose activity is controlled by a series of Rsb proteins encoded by *rsb* genes (*rsbUVWsigB*), *cspA* gene that encode CspA cold shock protein and *hfq* gene encoding Hfq protein that acts as a multifunctional regulator in bacteria (Xue et al. 2019). The tested agents may act differently on these genes in different isolates resulting in increased staphyloxanthin production in some isolates.

In this study, the effect of the tested agents on the downregulation of QS regulating genes *crtM, sigB, sarA, fnbA, agrA* and *icaR* was tested using qRT-PCR. It was found that the tested inhibitors significantly decreased the expression levels of *crtM*, *sigB*, *sarA* and *fnbA*, while significant increase was observed in the expression levels of *agrA* and *icaR* genes (Fig. 4). Among all tested inhibitors, Ag-MET-Ns was the most efficient one against QS controlled virulence genes, this indicates synergism interaction occurred between MET and AgNPs. The upregulation of *agrA* and *icaR* agreed with a previous study on biofilm formation by *S. epidermidis*, in which an agrC-specific binding polypeptide upregulated the expression of *atlE*, *icaA*, *fbe*, and *icaR* genes after 18 h of culture following a transient downregulation of these genes after 12 h of culture. In our study, the effect on gene expression was studied after overnight culture (Xiang et al. 2020). Moreover, Gheidar et al. (2018) found that AgNPs downregulated *fnbA* and *fnbB*, while no significant reduction was observed with *icaA* and *icaD* genes as compared to the untreated controls. Liang et al. (2019) reported downregulation of the expression levels of the genes encoding biofilm; *icaA*, *sarA* and *cidA* using 12-tungstophosphoric acid silver salt (Ag_3_PW_12_O_40_) nanoparticles (AgWPA-NPs). However, Singh et al. (2019) found no significant difference in virulence genes’ expression in both AgNPs and Ag^+^ treated biofilms in spite of the variation in gene expression as shown by fold change values. The variation in the effect of tested agents on different isolates may be also due to the difference in the sources of the isolates. It was previously reported that there is variation in virulence genes expression in strains of *Pseudomonas aeruginosa* isolated from different sources (Sameet et al. 2020).

The inhibitory activities of the tested inhibitors against *S. aureus* pathogenesis were determined in vivo, the highest protection was found with mice group injected with sub-MIC of MET-Nano; being the most effective among the tested inhibitors. In addition, Metformin nano showed much lower accumulation than the bulk metformin or metal nanoparticles, therefore reducing cytotoxicity occurring after exposure. Similarly, Li et al. (2020) showed that in an in vitro cytotoxicity experiments revealed that FTP NPs were less toxic to mammalian cells than PMET, and in vivo intravenous injection of FTP NPs revealed no acute toxicity. In another study reported by Liao et al. (2021), it was found that treatment with nanostructured lipid carriers (NLCs) encapsulating both ciprofloxacin and rolipram significantly reduced MRSA count in mice liver and kidneys compared with the free drugs. In addition, Mekkawy et al. (2017) observed much higher antibacterial activity, as well as wound healing promotion of Na CMC hydrogel loaded with PEG-coated AgNPs as compared to silver sulfadiazine cream using MRSA-infected wound mice model. In summary, *S. aureus* is a leading cause of life-threatening MDR infections worldwide. With shortage of new antibiotic development, novel approaches are required such as repurposing the FDA approved drugs against bacterial QS system. The oral hypoglycemic agent metformin exhibited better anti-QS activity against MDR *S. aureus* when it was nanoformulated. Its antibacterial and anti-QS activities were augmented when combined with silver nanoemulsion.

## Data Availability

The authors confirm that the data supporting the findings of this study are available within the article.
